# Postpartum Acute Pseudo-Obstruction of the Colon (Ogilvie's Syndrome) Leading to Perforation After Spontaneous Vaginal Delivery

**DOI:** 10.7759/cureus.72881

**Published:** 2024-11-02

**Authors:** Khadija Yawar, Hamza N Virk, M. Sohaib Ejaz, Nabiha N Rizvi, Wasif M Chaudhry

**Affiliations:** 1 Nephrology, Ghurki Trust and Teaching Hospital, Lahore, PAK; 2 Nephrology, Farooq Hospital, Lahore, PAK; 3 General Surgery, Ghurki Trust and Teaching Hospital, Lahore, PAK; 4 General Surgery, Lahore Medical and Dental College, Lahore, PAK; 5 General Surgery, Surgimed Hospital, Lahore, PAK

**Keywords:** colonic pseudo-obstruction, endoscopic decompression, neostigmine, ogilvie's syndrome, open cecostomy, perforated colon

## Abstract

Acute pseudo-obstruction of the colon (Ogilvie's syndrome) leading to perforation is a rare post-surgical complication. Most commonly associated with cesarean sections, only one case has been previously reported in the literature that occurred after normal vaginal delivery. We present the case of a 31-year-old female who presented on the seventh day following spontaneous vaginal delivery with abdominal distension, intermittent episodes of watery diarrhea, and sudden severe generalized abdominal pain. Radiological imaging showed evidence of pneumoperitoneum and perforation of the bowel. A right-sided hemicolectomy was performed after an exploratory laparotomy revealed perforation of the cecum. Initially considered a diagnosis of exclusion, Ogilvie's syndrome is a rare and multifactorial post-surgical complication. This case report emphasizes the significance of early detection and initiation of treatment, which can minimize the possibility of developing mortality-associated sequelae of this disease.

## Introduction

Ogilvie's syndrome is defined as acute pseudo-obstruction of the bowel without any mechanical obstruction in the presence of both clinical and radiological evidence. A rare postoperative phenomenon, it is characterized by the signs, symptoms, and radiological evidence of large bowel obstruction but without an organic cause [1,2]. At the moment, it has three modalities of treatment, conservative, pharmacological, and surgical, with the former two preferred over the latter due to the associated high risk of mortality. Various cases have linked cesarean section to acute pseudo-obstruction, but there is only one case previously reported that cites Ogilvie's syndrome as the cause of bowel perforation post-normal vaginal delivery [3]. Timely diagnosis and treatment of pseudo-obstruction are necessary to reduce the occurrence of mortality-associated sequelae.

## Case presentation

Following a spontaneous vaginal delivery at 32 weeks, a 31-year-old woman (parity 4 abortion 0) appeared on the seventh postnatal day with abdominal distension, diarrhea, and the sudden onset of severe right hypochondrium pain that eventually became generalized. The patient reported having infrequent episodes of pain in the right iliac fossa for the previous eight months and several episodes of watery, foul-smelling loose stools throughout the previous day. She complained of shortness of breath that started two hours before her admission with no prior history of coughing, dyspnea, orthopnea, paroxysmal nocturnal dyspnea, chest pain, or palpitations. There were no complaints about weight loss, night sweats, vomiting, constipation, or melena. There was no prior history of any rash, joint pain, headache, fits, or loss of consciousness. The patient had taken folic acid only in the first trimester, had her dating and anomaly scans done, and was perceiving normal fetal movements. There was no history of vaginal spotting, bleeding, or discharge. There were no urinary tract concerns, nor was there a history of hypertension or gestational diabetes.

At the time of presentation, the patient's vital signs were within normal limits, and she had passed both stool and flatus once within the past 24 hours. On examination, the abdomen was soft and tender, had a positive fluid thrill, and was distended with an abdominal girth measuring 104 cm with sluggish bowel sounds. Crepitations could be heard on left-sided chest auscultation. An urgent abdominopelvic scan was advised, along with urgent consultations by both Departments of Internal Medicine and General Surgery. They advised that the patient be kept nil per os, i.e., nothing by mouth (NPO), until further lab reports.

Her laboratory investigations showed leukocytosis (14.8×10^9^/L), metabolic acidosis (pH: 7.32 and HCO3: 9.8 mmol/L), raised serum creatinine and urea (1.5 mg/dl and 142 mg/dl, respectively), thrombocytosis (625×10^9^/L), anemia (hemoglobin levels at 8.8 g/dl), and hypoalbuminemia (2.1 g/dl). Her potassium and sodium levels were normal. Prothrombin time was 10 seconds, fibrinogen was 551 mg/dl, and D-dimer levels were 0.80 FEU/ml. Her viral markers for hepatitis B and C were negative. Urinalysis showed the presence of bacteria, so a urine culture was ordered. Abdominopelvic ultrasound showed moderate debrinous ascites within the abdomen for which an ascitic tap was done. There was no sonographic evidence of remaining products of conception, no heavy bleeding, or no presence of foul-smelling lochia, and the high vaginal swab culture and sensitivity that had been previously advised showed no growth. 

**Figure 1:**
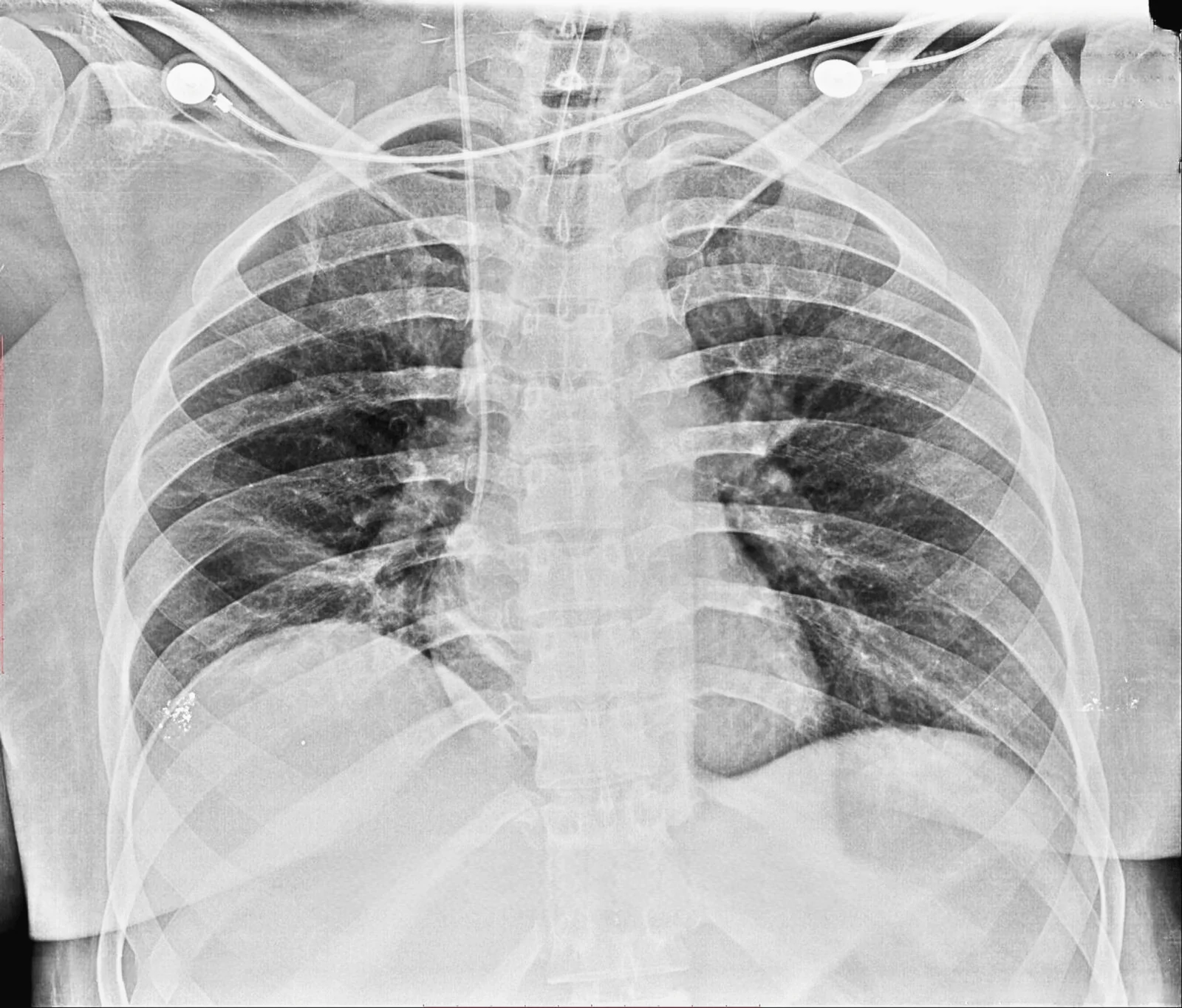
Portable ICU chest X-ray (supine, AP view). The image shows limited visualization of bilateral lung fields. No obvious gross abnormality is observed. ICU: intensive care unit; AP: anteroposterior

She was started on 1 g of intravenous (IV) ceftriaxone sodium (twice a day), 500 mg/100 ml of IV metronidazole (three times a day), 4.5 g of IV piperacillin+tazobactam (three times a day), and 1000 ml of normal saline with four ampules of bicarbonate infused at 120 ml/hr. On the second day of admission, the patient's abdominal distension worsened, she became increasingly disoriented and tachypneic (respiratory rate: 42/min), her skin was cold and clammy, and she had a randomly tested blood sugar level (BSR) of 64 mg/dl. Worsening of her clinical condition and her lab reports were now pointing towards septic shock, following which she was shifted to the intensive care unit (ICU) for strict monitoring and was given four ampules of 25% dextrose water and a packed cell volume (PCV) transfusion. Despite being on both ceftriaxone sodium and piperacillin+tazobactam, there was an increase in white blood cell count (from 14.8 to 29.08×10^9^/L); hence, both were discontinued. An IV dose of 1 g of meropenem was added instead. BSR repeated after half an hour was at 97 mg/dl. A portable chest X-ray performed while in the ICU showed no gross abnormalities (Figure 1).

**Figure 2:**
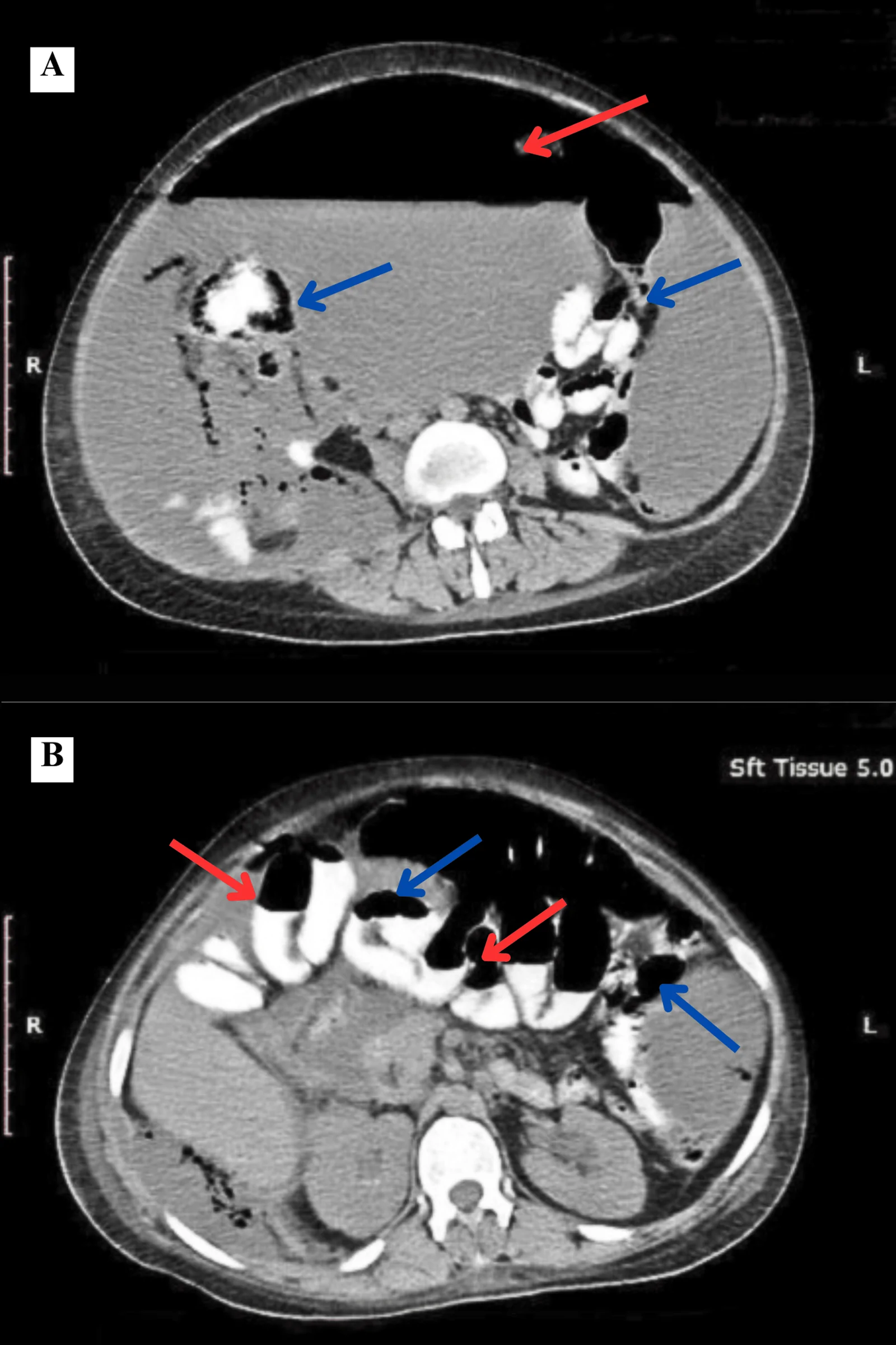
CT of the abdomen and pelvis axial view (oral and IV contrast). The image shows evidence of massive abdominopelvic ascites with large air-fluid levels (red arrows) and multiple air lucencies (blue arrows) representing pneumoperitoneum. IV: intravenous

CT of the abdomen and pelvis with both oral and IV contrast gave evidence of massive abdominopelvic ascites with pneumoperitoneum and seemed to indicate a likely site of perforation around hepatic flexure/ascending colon of the large bowel. It showed hyper-enhancement/thickening of collapsed hepatic flexure with suspiciously high density in the surrounding as well as ascending colon, suggesting extra-luminal contrast perforation around this region (Figure 2).

An exploratory laparotomy was performed on the fourth day of admission via a midline incision, which showed approximately a 4×4 cm perforation of the cecum with a mass/phlegmon involving the hepatic flexure, liver, and gallbladder with multiple interloop adhesions within the small bowel. The gallbladder containing stones was tightly adhered to the large bowel. Around 4.5 liters of fecal material was washed out, and 2 feet of the terminal ileum, the cecum, the ascending colon, the hepatic flexure, and the proximal two-thirds of the transverse colon were mobilized. The patient underwent a cholecystectomy, along with a right-sided hemicolectomy with the terminal ileum brought out as the end ileostomy and the transverse colon stump closed in two layers. The abdominal cavity was washed with 7 liters of normal saline, followed by the placement of a right subhepatic and pelvic drain. Polypropylene 1-0 sutures were used for the closure of the abdomen, but the skin was kept open.

Due to a rapid decline in the patient's postoperative vitals, i.e., blood pressure (BP) at 80/60 mmHg, heart rate (HR) at 126 bpm, respiratory rate at 27/min, blood sugar level at 293 mg/d, temperature at 98°F, spO2 99% at 80% FiO2 (fraction of inspired oxygen), and a sluggish light reflex, the patient was sedated and placed on mechanical ventilation with norepinephrine infused at 10 ml/hr. Postoperatively, central venous pressure (CVP) remained between 8 and 12 mmHg. Twelve hours post-op, the subhepatic drain contained 500 ml of serosanguineous fluid, and the pelvic drain contained 150 ml of serosanguineous fluid, while there was no output within the ileostomy bag. The patient was transfused with three pints of whole blood (one during the procedure and two postoperatively). She was started on IV tazobactam+piperacillin 4.5 g (three times/day).

On the first post-op day, reports of ascitic tap and urine culture were received, which, after 72 hours of incubation, were both positive for different strains of *Escherichia coli,* which was tested for drug sensitivity, and the patient was started on gentamicin. She remained sedated and paralyzed, was being given inotropic support, was hypotensive, and had decreased air entry at lung bases bilaterally. She was given multiple weaning off-trials, but all were unsuccessful. The wound appeared healthy without any discharge. There was a marked reduction of white blood cell (WBC) count (from 25.73 to 14.69×10^9^/L), but on the second post-op day, the patient developed respiratory alkalosis. Over the next few days, the patient, despite being on mechanical ventilation, made no improvements whatsoever.

**Figure 3:**
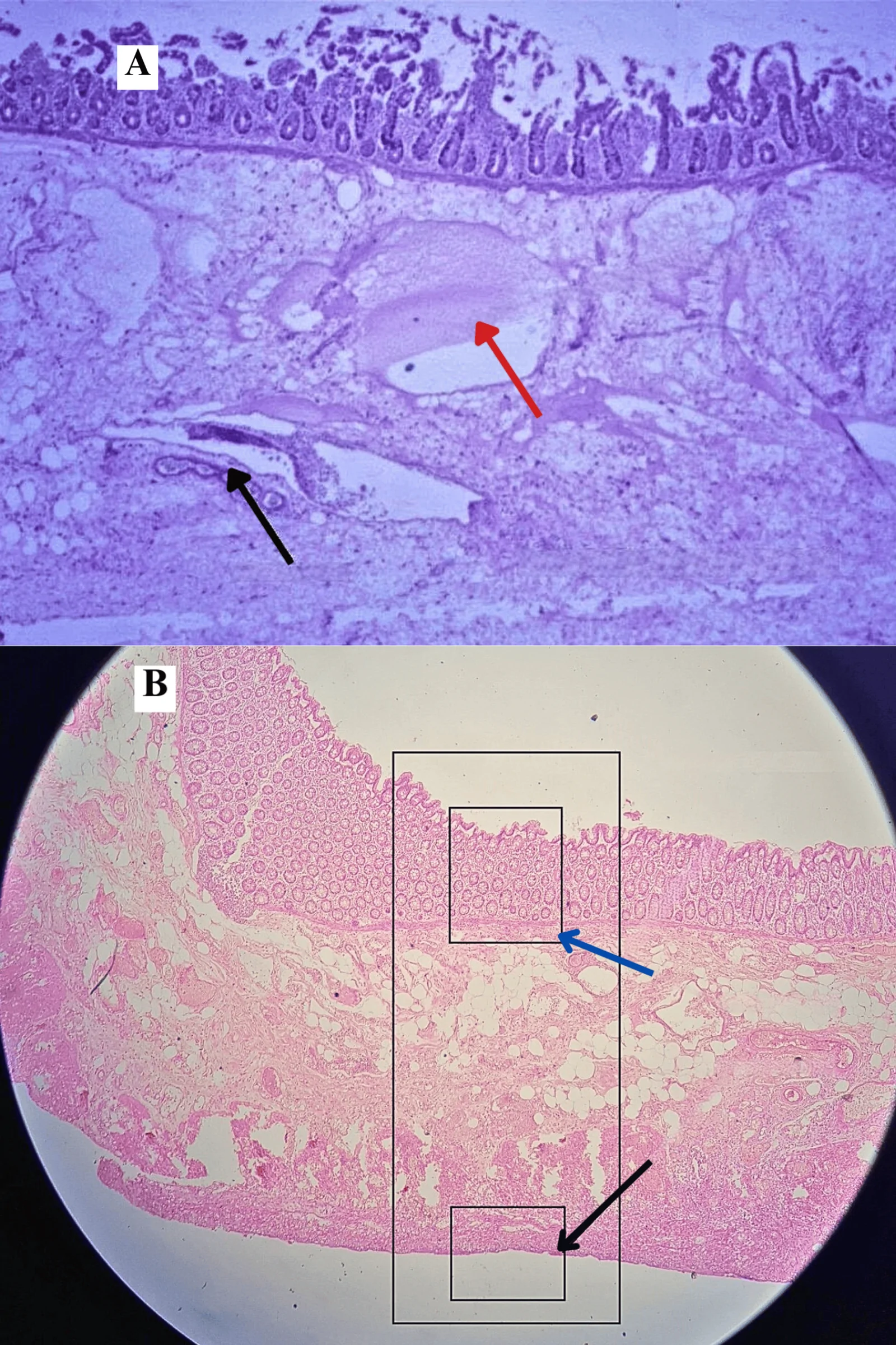
Microscopic examination of a section from the large bowel and colonic resection margin. A: Section from the large bowel: ischemic necrosis (red arrow) and vascular congestion (black arrow). B: Colonic resection margin: histological evidence of inflammation by the presence of an increased number of inflammatory cells at the serosal surface (black arrow). Mucosal surface represented by blue arrow.

**Figure 4:**
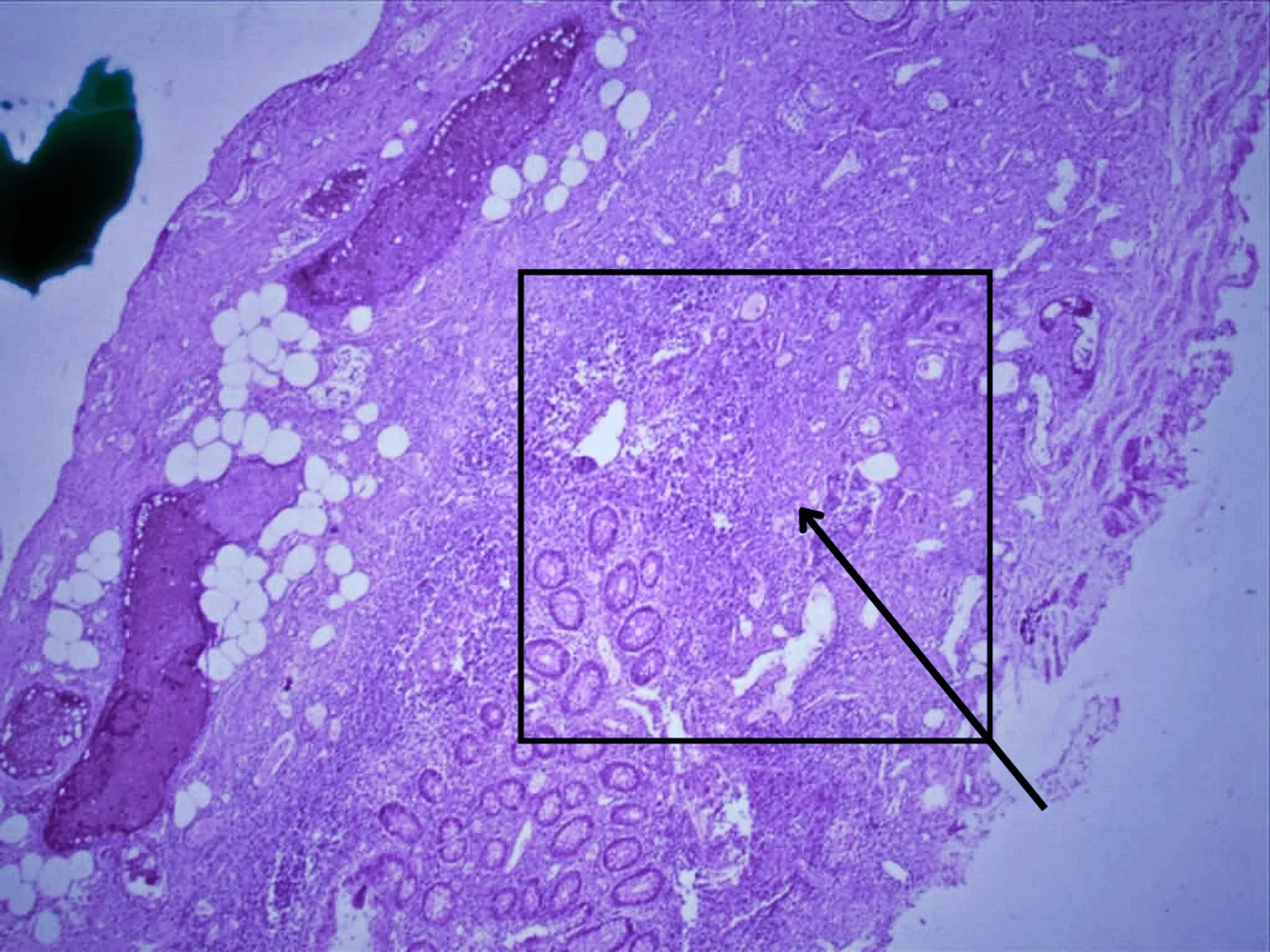
Microscopic examination of a section from the site of perforation. The image shows loss of surface epithelium, poorly cellular material, and loss of normal continuation of glands, features consistent with those of ulceration. There was no evidence of granulomas.

According to the biopsy report, grossly, the cecum appeared black with areas of mucosal hemorrhage. Histological evaluation of intestinal biopsy specimens revealed inflammation, serosal congestion, and fibroblastic reactions consistent with perforation within the mucosa of the large bowel. Ileal and colonic resection margins were inflamed. However, no evidence of granuloma or malignancy was seen, as evidenced by Figure 3 and Figure 4.

On the fifth post-op day, since multiple spikes of 100-101°F fever were recorded along with tachycardia (HR >150 bpm) and coarse crepitations could be heard on auscultation bilaterally, the patient was started on 500 mg of IV amikacin sulfate, which was to be given twice a day. Her C-reactive protein (CRP) levels were 110.43 mg/l (normal <5), and her blood culture report for 24 hours and after seven days of incubation showed no growth, potentially ruling out the diagnosis of typhoid. On the sixth post-op day, the patient's CRP levels were 129.89 mg/l. She suddenly developed bradycardia, and her saturation levels started dropping significantly. Despite multiple resuscitation efforts, the patient was unable to survive.

A summary of complete lab investigations carried out since the day of admission has been provided (Table 1, Table 2, Table 3, Table 4).

**Table 1:** Detailed summary of investigations since the day of admission (part 1): arterial blood gases, complete blood count, and chemistry panel. Hb: hemoglobin; MCV: mean corpuscular volume; ALP: alkaline phosphatase; HCO3: bicarbonate; pO2: partial pressure of oxygen; pCO2: partial pressure of carbon dioxide; RBC: red blood cells; WBC: white blood cells

Investigation		Reference range	Day since admission										
			1	2	3	4 (pre-op)	4 (post-op)	5	6	7	8	9	10 (sixth day post-op)
Arterial blood gases													
pH	7.35-7.45		7.2	7.38	7.4	7.4	7.33	7.37	7.51	7.33	7.31	7.25	7.2
pCO2 (mmHg)	35-45		19	17	18	18	24	25	17	29	28	29	42
pO_2_ (mmHg)	83-108		53	154	172	128	190	132	200	80	61	153	82
HCO_3_ (mmol/l)	21-28		9.8	10.1	11	11.1	12.7	14.5	13.6	15.3	14.1	12.7	16.4
O_2 _saturation (%)	95-98		84	99	100	99	100	99	100	95	90	99	93
Complete blood count													
WBC (×10^9^/L)	4-11		14	29	21	-	34.2	14.7	15.3	19.1	14.6	14.2	16.3
RBC (×10^12^/L)	4.5-6.5		3.7	4.2	4.7	-	4.49	3.9	4.02	4.82	4.77	4.49	4.26
Hb (g/dl)	12-16		8.8	10.5	11	-	11.9	10.7	11	12.8	13.3	12.8	11.6
Platelet count (×10^9^/L)	150-400		625	573	521	-	345	229	230	235	168	343	445
MCV (fL)	75-95		72	74.4	75	-	80.6	83.5	83.6	83.6	85.5	84.6	87.6
Hematocrit (%)	38-52		27	31.6	35	-	36.2	32.6	33.5	40.3	40.8	38	37.3
Chemistry													
Sodium (mmol/l)	135-153		133	139	144	141	-	149	147	-	150	150	140
Potassium (mmol/l)	3.3-5.1		3.6	4	4	5	-	4.2	4.6	-	3.8	3.5	-
Creatinine (mg/dl)	0.6-1.4		1.5	1.2	1.2	1.4	-	1.2	1.2	-	0.8	0.7	0.6
Urea (mg/dl)	10-50		142	129	132	129	-	116	90	-	68	40	25
Total bilirubin (mg/dl)	<1.1		0.6	-	0.7	1.1	-	0.6	-	-	0.7	0.5	-
ALP (U/L)	70-300		296	-	345	361	-	292	-	-	1432	949	-
Total protein (g/dl)	6-8.3		5.1	-	6	5	-	5.5	-	-	5	5	-
Albumin (g/dl)	3.5-5.5		2.1	-	2.5	2.5	-	2.5	-	2.6	2.5	2.5	-
A/G ratio	1.4-1.8		0.7	-	0.7	1	-	0.83	-	-	1	1	-
Calcium (mg/dl)	8.4-10.2		-	-	-	-	-	-	-	-	-	8.1	8.1

**Table 2:** Detailed summary of investigations since the day of admission (part 2): hepatitis serology. HBsAg: hepatitis B surface antigen; HCV: hepatitis C virus; HAV: hepatitis A virus; HEV: hepatitis E virus; Anti-HCV, Anti-HAV, Anti-HEV: antibody tests

Hepatitis serology		
HBsAg	Negative	
Anti-HCV	Negative	
Anti-HAV IgM	Negative	
Anti-HAV IgG	Negative	
Anti-HEV IgG	Negative	
Anti-HEV IgM	Negative	

**Table 3:** Detailed summary of investigations since the day of the admission (part 3): culture and sensitivity.

Culture and sensitivity				
Specimen	Result			
Urine	*Escherichia coli* (viable count 10^4^)	Sensitive to amikacin, fosfomycin, minocycline, nitrofurantoin, colistin, gentamicin		
Ascitic fluid	Escherichia coli	Sensitive to co-amoxiclav, doripenem, minocycline, tobramycin, polymyxin B, colistin, ertapenem, cefepime, cefoperazone/sulbactam, meropenem, chloramphenicol, gentamicin, piperacillin+tazobactam, amikacin, imipenem		
Blood	No growth after 24 hours and seven days' incubation			

**Table 4:** Detailed summary of investigations since the day of admission (part 4): coagulation profile. PT: prothrombin time; INR: international normalized ratio; aPTT: activated partial thromboplastin time

Coagulation profile				
	Normal range	Day of admission		
		2	4	5
PT	11-16 seconds	10	12	11
Control PT		10	10	10
INR (calculated value)		1	1.2	1.1
APTT	30-40 seconds	30	30	33
Control APTT		30	30	30
Fibrinogen	200-400 mg/dl	551	-	-

## Discussion

Acute pseudo-obstruction of the colon, also known as Ogilvie's syndrome, is characterized by the massive dilation of the colon without any evidence of mechanical obstruction. First described in the literature by Sir William Heneage Ogilvie in 1948 [4], this syndrome, according to varying hypotheses, is believed to be caused by an imbalance between the motor and autonomic innervation of the colon leading to colonic dilation mostly involving the cecum and the ascending colon. Although this disease is primarily a cause for concern in elderly individuals, the most common causative factors in young people, especially women, include cesarean section, trauma, and/or pelvic surgery [5]. Other theories suggest that overstimulation of sympathetic or inhibition of parasympathetic activity, i.e., loss of activity in areas supplied by the vagus nerve and especially those supplied by the pelvic splanchnic nerves, may lead to acute colonic dilation resulting in hypotonic distal colon. 

Theories regarding the etiology of this condition include hormonal theory, disturbances in peristaltic activity stimulated by prostaglandin E; vascular theory, hypoperfusion of the bowel due to ineffective mesenteric circulation; pharmacological theory, linking calcium channel blockers, anti-Parkinsonian medications, and opioids to their toxic effects; and a metabolic theory linking various metabolic disorders such as electrolyte disturbances to neuromuscular dysfunction [6]. As reported by Strecker and Jaluvka, decreasing serum levels of estrogen in the postpartum period may be the main link between Ogilvie's syndrome and normal vaginal delivery [7]. Due to a lack of understanding regarding its pathophysiology, as only one case has been previously mentioned in the literature, the exact incidence of this condition is unknown. However, one report suggests the incidence of postpartum Ogilvie's syndrome is one out of every 1500 deliveries [8]. This rare and poorly understood condition carries a high mortality rate, with the most important potential sequelae being perforation of the large bowel, followed by peritonitis, sepsis, and eventually death. 

The most common presentations in the 125 cases reported postpartum were nausea, vomiting, abdominal distension, and absolute constipation, with some patients presenting with infrequent episodes of fever as well. Other frequently encountered findings may include dyspnea, which occurs as a consequence of increasing abdominal distension [9]. The diagnosis of acute pseudo-obstruction of the colon is mainly clinical. Since it can either rapidly progress within 24-48 hours or take five to seven days to occur in the postoperative period, it should not be ruled out as a high possibility. A detailed physical examination and an assessment of a patient's hemodynamic stability play a crucial role in making a diagnosis. Acute pseudo-obstruction is a diagnosis of exclusion; hence, it is necessary to rule out all possibilities of a mechanical obstruction before making a confirmation. In the case discussed above, the main differential diagnoses for this patient were either typhoid, tuberculosis, puerperal sepsis, or spontaneous bacterial peritonitis, the possibilities of which were ruled out after specific pathological and radiological investigations. Other possible diagnoses include ischemic colitis, toxic megacolon, and colonic malignancies, which should be excluded based on different imaging modalities and histological examination of biopsy specimens.

The presence of air under the diaphragm and multiple air-fluid levels on an erect abdominal X-ray supports the diagnosis of perforation as evidenced by the radiograph attached. CT of the abdomen and pelvis is more effective than a contrast enema in providing evidence of pneumoperitoneum and sites of perforation [10]. Leukocytosis, elevation of CRP, and metabolic acidosis as evidenced by the arterial blood gases combined with the radiological investigations provide an appropriate estimation of the patient's condition and early recognition and initiation of treatment, which can lead to a better prognosis. The conservative method of treatment is the preferred method of choice, especially in cases where there is no immediate risk of perforation. It needs to be initiated as early as possible considering the high mortality associated with this condition. 

Conservative methods include the correction of underlying metabolic abnormalities such as hypocalcemia, hypokalemia, and hypomagnesemia, which significantly contribute to alterations in colonic motility as a result, predisposing patients to the risk of pseudo-obstruction. Other methods include the use of prokinetic agents such as erythromycin or metoclopramide to enhance gut motility or using mild laxatives, besides osmotic laxatives, to facilitate decompression of the colon [11]. Conservative measures involve discontinuation of any anti-peristaltic pharmacological agents, fluid resuscitation, and nasogastric tube decompression. Colonic decompression can also be achieved either through the use of endoscopic methods or via a bolus dose of 2 mg of neostigmine followed by a continuous infusion, which, although has a success rate of 91%, carries an increasing risk of recurrence, eventually requiring extensive surgical interventions. Neostigmine, an acetylcholinesterase inhibitor, increases the levels of acetylcholine, which stimulates a parasympathetic response and initiates peristalsis, thereby decreasing colonic dilation. The use of neostigmine carries its risks, with patients developing cholinergic side effects such as bradycardia and bronchospasm and thus requiring extensive cardiac monitoring, especially in patients with renal impairment where the half-life of the administered drug is increased. Therefore, it is required either that neostigmine be administered in a slow, continuous IV infusion, i.e., 0.4-0.8 mg/hour for 24 hours, or that the initial dose does not exceed 1 mg [12].

The use of polyethylene glycol is recommended after the resolution of Ogilvie's syndrome to decrease the rate of recurrence. In a randomized study trial, patients who received polyethylene glycol after neostigmine decompression showed no recurrence compared to a 33% recurrence rate in the placebo group [13]. Endoscopic placement of a soft tube in the rectum or cecum under fluoroscopic guidance is suggested for those who fail pharmacological therapy. Different procedures for endoscopic catheterization of the colon have been explained by Harig et al. [14] and Groff [15], with varying rates of success. Surgical cecostomy is an intervention highly indicated in patients who are unresponsive to other therapies. Percutaneous cecostomy or percutaneous endoscopic cecostomy is an intervention preferred over tube cecostomy due to the potential complications associated with the former, such as pressure necrosis, tube occlusion, and granulation tissue formation. Based on the patient's rapidly deteriorating state and the possibility of ineffective conservative therapy, a surgical approach was considered in the abovementioned case. According to the guidelines of the American Society of Colon and Rectal Surgeons, in the absence of a raised leukocyte count, fever, or cecal diameter >12 cm, first-line management includes correcting underlying abnormalities contributing to the patient's condition [16]. Surgical management is reserved for hemodynamically unstable patients with signs of perforation or ischemia requiring prompt management [17]. Each surgical intervention is tailored to the patient's condition, associated comorbidities, and if the benefits of management outweigh the risks, i.e., 30-44% mortality [18-20].

## Conclusions

Ogilvie's syndrome is a rare but high mortality-associated complication of spontaneous vaginal delivery. There is not enough data regarding the incidence of this condition, as it is most commonly linked to cesarean sections; however, increasing unexplained abdominal distension and pain postpartum should raise a clinical suspicion of this diagnosis. Ogilvie's syndrome poses a great challenge, as without early diagnosis and treatment, life-threatening sequelae such as large bowel perforation, fecal peritonitis, and maternal mortality can occur eventually, leading to extensive surgical interventions that pose great risks of their own. Conservative measures, especially the use of neostigmine and colonic decompression through endoscopy, are the preferred methods of treatment. Surgical treatment is considered in patients in whom these measures are ineffective and acute pseudo-obstruction of the colon is complicated by either perforation or ischemia.
