# Correction: Postpartum Acute Pseudo-Obstruction of the Colon (Ogilvie's Syndrome) Leading to Perforation After Spontaneous Vaginal Delivery

**DOI:** 10.7759/cureus.c490

**Published:** 2026-10-13

**Authors:** Khadija Yawar, Hamza N Virk, M. Sohaib Ejaz, Nabiha N Rizvi, Wasif M Chaudhry

**Affiliations:** 1 Nephrology, Ghurki Trust and Teaching Hospital, Lahore, PAK; 2 Nephrology, Farooq Hospital, Lahore, PAK; 3 General Surgery, Ghurki Trust and Teaching Hospital, Lahore, PAK; 4 General Surgery, Lahore Medical and Dental College, Lahore, PAK; 5 General Surgery, Surgimed Hospital, Lahore, PAK

Paragraph 5 of the Discussion section has been corrected to state that the neostigmine bolus dose is 2 mg. This was previously incorrectly reported as 2 g.

